# Fuzzy Decision Making Approach to Identify Optimum Enzyme Targets and Drug Dosage for Remedying Presynaptic Dopamine Deficiency

**DOI:** 10.1371/journal.pone.0164589

**Published:** 2016-10-13

**Authors:** Kai-Cheng Hsu, Feng-Sheng Wang

**Affiliations:** Department of Chemical Engineering, National Chung Cheng University, Chiayi 62102, Taiwan; Tianjin University, CHINA

## Abstract

Model-based optimization approaches are valuable in developing new drugs for human metabolic disorders. The core objective in most optimal drug designs is positive therapeutic effects. In this study, we considered the effects of therapeutic, adverse, and target variation simultaneously. A fuzzy optimization method was applied to formulate a multiobjective drug design problem for detecting enzyme targets in the presynaptic dopamine metabolic network to remedy two types of enzymopathies caused by deficiencies of vesicular monoamine transporter 2 (VMAT2) and tyrosine hydroxylase (TH). The fuzzy membership approach transforms a two-stage drug discovery problem into a unified decision-making problem. We developed a nested hybrid differential evolution algorithm to efficiently identify a set of potential drug targets. Furthermore, we also simulated the effects of current clinical drugs for Parkinson’s disease (PD) in this model and tried to clarify the possible causes of neurotoxic and neuroprotective effects. The optimal drug design could yield 100% satisfaction grade when both therapeutic effect and the number of targets were considered in the objective. This scenario required regulating one to three and one or two enzyme targets for 50%–95% and 50%–100% VMAT2 and TH deficiencies, respectively. However, their corresponding adverse and target variation effect grades were less satisfactory. For the most severe deficiencies of VMAT2 and TH, a compromise design could be obtained when the effects of therapeutic, adverse, and target variation were simultaneously applied to the optimal drug discovery problem. Such a trade-off design followed the no free lunch theorem for optimization; that is, a more serious dopamine deficiency required more enzyme targets and lower satisfaction grade. In addition, the therapeutic effects of current clinical medications for PD could be enhanced in combination with new enzyme targets. The increase of toxic metabolites after treatment might be the cause of neurotoxic effects of some current PD medications.

## Introduction

Parkinson's disease (PD) is a chronic and progressive neurodegenerative disorder and is the most common movement disorder, affecting more than 1% of the population aged more than 65 years worldwide [1–4]. PD is mainly characterized by a progressive loss of dopamine neurons in the pars compacta of the substantia nigra, and a loss of dopamine neurons in the extrapyramidal system contributes to the motor symptoms of PD. Consequently, the treatment options for PD have been focused on restoring the dopamine function by replacing dopamine precursors and agonist or inhibiting dopamine degradation. Several drugs affecting enzymes involved in dopamine metabolism have been used for treating PD. For many years, L-3,4-dihydroxyphenylalanine (L-DOPA) has been administered for treating PD symptoms. However, whether L-DOPA exacerbates PD because of L-DOPA oxidation and side products has been debated [5]. By contrast, the deprenyl and tocopherol antioxidative therapy of Parkinsonism (DATATOP) study and other follow-up trials have demonstrated that monoamine oxidase inhibitor (MAOI) delays the use of L-DOPA [6–9] and reduces the rate of motor fluctuations [10]. Such observations indicate that the treatment of PD has to consider therapeutic and adverse effects simultaneously.

The process of making a new medicine is a challenging and endurance task [11–12]. Recent advances in molecular medicine and powerful tools to enhance computational capacity are enabling researchers to better understand the inner workings of human disease at the molecular level. Model-based optimization methods are recently applied to the early drug discovery process [13–15]. This study introduces a fuzzy decision-making approach to screen candidate targets in the early stage of drug discovery process. The approach is a model-based optimization method which can include multiple objectives in the optimization problem. Such a drug discovery process may involve conflicting specifications, making it a challenging multiobjective optimization problem where numerous pharmaceutically crucial objectives must adequately be satisfied [16, 17]. A drug discovery problem is characterized by vast, complex solution spaces further perplexed by the presence of conflicting objectives. Mathematical modeling and optimization are the emerging technologies in drug development for human metabolic disorders [18–22]. Most optimal drug designs consider yielding positive therapeutic effects as the design specification; however, production of toxic metabolites after drug usage causes adverse effects. Therefore, achieving positive therapeutic effects and avoiding adverse effects must be simultaneously considered in drug design problems. Moreover, using the systems approach, identifying drug targets with high enzyme activities and low drug doses to minimize adverse effects is essential. The minimum effective dose (MED) is the lowest dose level of a pharmaceutical product that provides a clinically significant response with average efficacy that is significantly superior to the response provided by a placebo [23]. In this study, minimizing the variations of the identified enzymes, equivalent to MED, is considered a representative of the low-dose objective.

## Materials and Methods

### Optimization formulation

**Fig 1:**
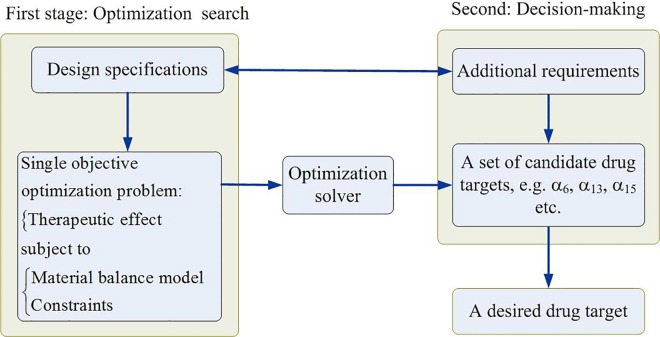
A two-stage optimization procedure to discover a new drug target. In the first stage, a designer assigns the therapeutic effect as the single objective in the optimization problem to obtain an optimal candidate target, and then to alter series requirements to be repeatedly resolved the problem to obtain a set of candidate targets. In the second stage, the designer considers some additional requirements to carry out a decision-making procedure for selecting a desired drug target among the candidates.

Most of optimal drug discovery problems use the therapeutic effect as the key criterion. A two-stage procedure shown in Fig 1 is a common approach to discover a new target, such as the optimization program for drug design (OPDD) to identify enzyme targets for remedying hyperuricemia [21, 22]. The first stage in Fig 1 is that a designer assigns a specification, e.g. therapeutic effect, and then such a specification is applied to formulate an optimization problem with constraints. An optimization solver is then applied to solve the single objective problem to obtain an optimal solution. The design is then altered a series of requirements, and then the problem is resolved repeatedly in order to obtain a set of targets. The second stage is a decision-making procedure wherein the designer uses some additional criteria, such as adverse effect and lower drug dose, to make a decision for selecting a desired drug target among the candidates.

**Fig 2:**
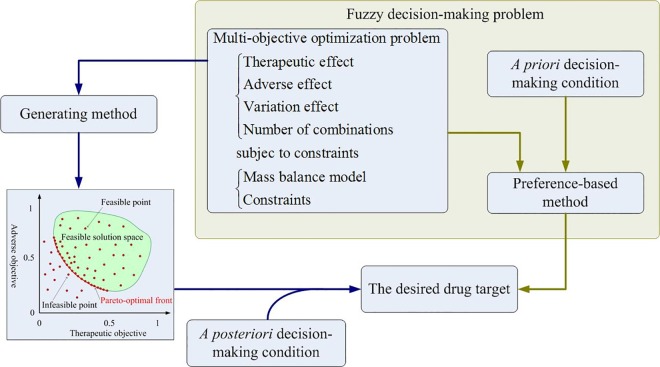
Computational procedures for multiobjective drug discovery problems. The left flowchart of the figure explains *a posteriori* decision-making method. A generating method is applied to yield a Pareto front of the MOO problem. For instance, the Pareto front for a two objective optimization problem is shown as the red curve. Some additional requirements are applied to make a decision for selecting a desired drug target from the candidate targets of the Pareto front. The green block diagram of the figure explains *a priori* decision-making design that the drug target discovery problem is formulated as a fuzzy multiobjective optimization problem. The decision-making conditions, such as a membership function for each objective, are included into the problem, and then a preference-based method is applied to obtain the desired drug target.

The criteria in the first stage and second stage can be combined together to formulate as a multiobjective drug discovery problem shown in Fig 2. The mathematical formulation of the multiobjective drug discovery problem is a type of multiobjective optimization (MOO) problems. Many methods are available for solving MOO problems, and each method has advantages and disadvantages [16, 17, 24, 25]. These methods are classified into two categories: generating and preference-based methods. Generating methods are applied for yielding a Pareto front of the MOO problem (left-hand side of Fig 2), where each point of the Pareto front is an optimal candidate of the MOO problem. Designers use decision-making criteria for performing a trade-off procedure to obtain the desired optimal target. Such methods are referred as *a posteriori* decision-making method. Preference-based methods are applied to solve problem wherein designers have certain expectations of the optimal solution; in this scenario, a *priori* condition can be implemented along with the preference-based method. Such a trade-off procedure is then applied in the priori decision-making method to obtain the desired target.

This study introduces a fuzzy decision-making method for formulating the aforementioned preference-based method into a fuzzy multiobjective drug discovery (FMDD) problem as shown in the green box of Fig 2. The mathematical formulation of the FMDD problem is expressed as follows:

$$\begin{array}{l} \text{Objectives}:\\ \left\{ \begin{array}{l} \underset{x,{\hat{\alpha }}_{j},u_{j,}z_{j}}{Fuzzy\;equal}\;x_{i}\approx x_{i}^{HS};\;i\in \Omega^{TE}\\ \underset{x,{\hat{\alpha }}_{j},u_{j},z_{j}}{Fuzzy\;\min}\;x_{j};\;j\in \Omega^{AE}\\ \underset{x,{\hat{\alpha }}_{j},u_{j},z_{j}}{Fuzzy\;equal}\;\alpha_{k}\approx \alpha_{k}^{basal};\;k\in \Omega^{VE}\\ \underset{x,{\hat{\alpha }}_{j},u_{j},z_{j}}{Fuzzy\;equal}\;u_{k}\approx u_{k}^{basal};\;k\in \Omega^{VE}\\ \underset{x,{\hat{\alpha }}_{j},u_{j},z_{j}}{Fuzzy\;\min}\;u_{l};\;l\in \Omega^{VE}\\ \underset{x,{\hat{\alpha }}_{j},u_{j},z_{j}}{\min}\sum z_{j} \end{array}\right. \end{array}$$ (1)

$$\begin{array}{l} \text{Equality constraints}:\\ \left\{ \begin{array}{l} \text{Kinetic model}:\\ \sum_{j=1}^{n_{v}}N_{ij}v_{j}+\sum_{j=1}^{n_{u}}B_{ij}u_{j}=0,i\in \Omega^{Species}\\ w_{j}={\hat{\alpha }}_{j}+\sum_{k=1}^{n_{v}}g_{jk}y_{k},j\in \Omega^{rxn}\\ v_{j}=\exp\left(w_{j}\right)\;(\text{nonlinear equation})\\ x_{i}=\exp\left(y_{i}\right)\;(\text{nonlinear equation})\\ \text{Disease restriction}:\\ {\hat{\alpha }}_{j}={\hat{\alpha }}_{j}^{DS},j\in \Omega^{DS} \end{array}\right. \end{array}$$ (2)

$$\begin{array}{l} \text{Inequality constraints}:\\ \left\{ \begin{array}{l} x_{i}^{LB}\leq x_{i}\leq x_{i}^{UB};\;i\in \Omega^{Species}\\ z_{j}{\hat{\alpha }}_{j}^{LB}+(1-z_{j}){\hat{\alpha }}_{j}^{basal}\leq {\hat{\alpha }}_{j}\leq z_{j}{\hat{\alpha }}_{j}^{UB}+(1-z_{j}){\hat{\alpha }}_{j}^{basal};j\in \Omega^{VE}\\ z_{k}u_{k}^{LB}+(1-z_{k})u_{k}^{basal}\leq u_{k}\leq z_{k}u_{k}^{UB}+(1-z_{k})u_{k}^{basal};k\in \Omega^{VE} \end{array}\right. \end{array}$$ (3)

The first objective in Eq (1) uses a fuzzy equal operation to evaluate therapeutic effects, where Ω^TE^ is the set of metabolites to be evaluated. For example, this study is to consider the dopamine level is as close to the healthy level as possible. Fuzzy minimization is applied in the second objective to evaluate the adverse effects of a set of metabolites Ω^AE^ that the targets can achieve to lower side effects. The third fuzzy equal is used to evaluate variation effects for the regulated enzyme targets, and the fourth and fifth objectives use fuzzy equal and fuzzy minimization to compute variation effects of external controls, where Ω^VE^ is the set of target enzymes and external controls excluding a set of disease enzymes Ω^DS^. The variation effect is applied to evaluate that the targets are regulated as small perturbation to their normal levels as possible. This objective means that we would like to find smaller change of the identified enzyme activity in order to achieve the therapeutic and adverse effect minimization goals. The external controls are considered as the regulable independent variables in the metabolic network. In general, we are difficult to use only one target to achieve all objectives as mentioned above. Some combinations of targets can be used to fulfill the requirements. The last objective is to consider the total number of the identified targets as less as possible.

### Kinetic model

The material balance model in Eq (2) can be generically governed by a set of nonlinear differential equations with the following structure:

$$\frac{dx}{dt}=Nv(x,\alpha )+Bu$$ (4)

where **x** ∊ R^n^ is a vector of n-dimensional metabolite concentrations or pools, *α* ∊ R^r^ is a vector of the r-dimensional enzyme activities, **v** ∊ R^r^ is a vector of reaction rates, **N** ∊ R^n×r^ is the stoichiometric matrix describing the interconnecting fluxes, **u** ∊ R^m^ is a vector of m-dimensional external controls, and **B** ∊ R^n×m^ is the connectivity matrix describing the interaction between a metabolite and its corresponding control. The connectivity matrix is similar to the stoichiometric property of a network. The model in Eq (4) with the external controls is a more general formulation, which differs from our previous study [26]. The reaction rate in this study is expressed according to the power law function described in Eq (5).

$$v_{k}=\alpha_{k}\prod_{l=1}^{n}x_{l}^{g_{kl}}$$ (5)

where *α*_*k*_ is the rate constant or enzyme activity for the *k*^*th*^ reaction rate and *g*_*kl*_ is the kinetic order. The power law function is based on the law of mass action in chemistry and biochemistry to express a mathematical model of a reaction rate [27, 28]. It has several benefit to formulate a biological system [27, 28], but is a nonconvex constraint in the MOO problem (2) that may result in a computational failure when the concentration approaches zero. We use the logarithmic expression for each rate, *w*_*k*_, and for each rate constant, ${\hat{\alpha }}_{k}$, in Eq (2) to prevent this numerical problem. The reaction rate and concentration are computed through the exponential operation. The advantage of this formulation is that the equality and inequality constraints in the optimization problem form a convex domain.

### Solving strategy

To solve the FMDD framework, we defined a membership function for each fuzzy equal objective and fuzzy minimizing objective for quantifying each corresponding satisfaction grade. The generalized membership function for each fuzzy equal objective is described in Eq (6) as follows:

$$\eta_{i}(x_{i})=\left\{ \begin{array}{l} 0,\;x_{i}\leq x_{i}^{LB}\\ d_{i}^{xL},\;x_{i}^{LB}\leq x_{i}\leq x_{i}^{basal,LB}\\ 1,\;x_{i}^{basal,LB}\leq x_{i}\leq x_{i}^{basal,UB}\\ d_{i}^{xR},\;x_{i}^{basal,UB}\leq x_{i}\leq x_{i}^{UB}\\ 0,\;x_{i}\geq x_{i}^{UB} \end{array}\right.$$ (6)

The left-hand side membership function is a strictly monotonically increasing function, $d_{i}^{xL}$, whereas the right-hand side is a strictly monotonically decreasing function, $d_{i}^{xR}$. A membership function is similar to assess the effects of inaccuracies in control variables and independent variables [29]. Monte Carlo simulation is generally applied to assess such an experimental imprecision. However, a designer can define a membership function for fuzzy optimization problems in advance. Sakawa [30] proposed five types of membership functions, namely linear, exponential, hyperbolic, inverse, and piecewise linear functions, for quantifying the behavior of fuzzy objectives or the constraints. Here, $x_{i}^{LB}$ and $x_{i}^{UB}$ are the lower and upper bounds of the *i*^th^ metabolite concentration or enzyme activity provided by the designer. The satisfaction grade is zero when the metabolite concentration or enzyme activity is beyond its lower or upper bounds. The satisfaction grade or membership function value is equal to one when the corresponding metabolite concentration or enzyme activity is between the lower and upper bounds of the basal value, represented as $x_{i}^{basal,LB}$, $x_{i}^{basal,UB}$, and $x_{i}^{basal}$, respectively. The satisfaction grade is between zero and one when the metabolite concentration or enzyme activity is within its range on the left- or right-hand side membership function. For each fuzzy minimizing objective, the membership function is defined as a strictly monotonically decreasing function on the right-hand side.

$$\eta_{j}(x_{j})=\left\{ \begin{array}{l} 1,\;x_{j}\leq x_{i}^{basal}\\ d_{i}^{xR},\;x_{i}^{basal}\leq x_{i}\leq x_{i}^{UB}\\ 0,\;x_{i}\geq x_{i}^{UB} \end{array}\right.$$ (7)

According to the membership functions expressed in Eqs (6) and (7), we conclude that the intersection for these membership functions is zero when either the fuzzy equal objective functions are outside the corresponding lower and upper bounds or the fuzzy minimizing objectives are greater than the corresponding upper bounds. By contrast, when all objectives are within their corresponding bounds, the intersection for all membership functions should show a certain degree of satisfaction. For each membership function being introduced by the designer, the FMDD problem is designed for determining a maximum intersection for all membership functions between the desired bounds. The FMDD framework is then transferred to the maximizing decision framework, which is a discontinuous function. The detailed procedures have been discussed previously [26, 30, 31]. Thus, the maximizing decision problem can be rewritten as an equivalent optimization problem on the solving domain to avoid a discontinuous computation, as follows:

$$\begin{array}{l} \underset{\lambda ,(x,\hat{\alpha },u,z)\in \Psi }{\max}\left(\frac{Z^{UB}-\sum z_{j}}{Z^{UB}-1}\right)\lambda \\ \eta_{i}\left(x_{i}\right)\geq \lambda ,\;i\in \Omega^{TE}\\ \eta_{j}\left(x_{j}\right)\geq \lambda ,\;j\in \Omega^{AE}\\ \eta_{k}\left({\hat{\alpha }}_{k}\right)\geq \lambda ,\;k\in \Omega^{VE}\\ \eta_{l}\left(u_{l}\right)\geq \lambda ,l\in \Omega^{VE} \end{array}$$ (8)

where the crisp feasible domain ψ includes the kinetic model and inequality constraints described in Eqs (2) and (3), respectively. The total number of the identified enzyme targets is the crisp objective so that it is transformed to a normalized value as expressed in Eq (8), where Z^UB^ is the upper bound. The advantage of this method is that the optimal membership grade corresponds to the satisfaction level for each objective, and the optimal decision λ represents the overall satisfaction grade (equivalent to the lower bound) of the problem.

### Nested hybrid differential evolution

The maximizing decision problem (8) is a mixed-integer nonlinear programming (MINLP) problem, which is typically difficult for solving to obtain a global solution. In general, MINLP problems are highly nonlinear and non-differentiable because of the combinatorial nature of the associated integer-valued variables. Conventional methods widely used to solve MINLP problems include the cutting plane, branch-and-bound, and decomposition methods and their variants [32–34]. These methods have been successfully applied to many practical problems; however, they require a suitable starting point and gradient information. Wu *et al*. [31] applied mixed-integer hybrid differential evolution (MIHDE) for identifying an enzyme intervention problem in metabolic networks; however, it was suitable only for low-dimensional problems. The computational time increased significantly when MIHDE was applied to a high-dimensional MINLP problem. Wang and Wu [35] proposed a nested hybrid differential evolution (NHDE) method for solving multilevel optimization problems derived to design mutant strain of genome-scale metabolic networks. This study modifies the NHDE algorithm for solving the MINLP problem.

**Fig 3:**
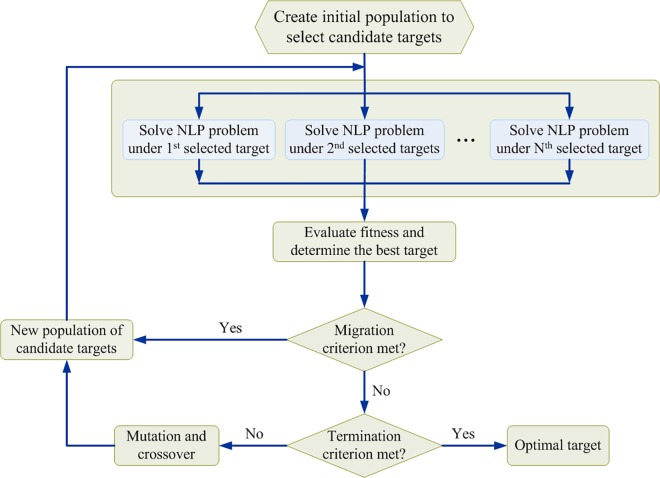
Flowchart of the modified algorithm for nested hybrid differential evolution. The core procedure of the NHDE algorithm is the evaluation and selection operation as shown in the second and third block diagram of the flowchart. The evaluation step is to solve each nonlinear programming (NLP) problem produced from the maximizing decision problem for each target candidate. The fitness of each NLP problem is computed for selecting the better individuals in the population, and then to generate the next individuals.

The basic operations of MIHDE are similar to those of the modified NHDE algorithm, and the flowchart of the modified NHDE algorithm is shown in Fig 3. The core procedure of modified NHDE is the integer variable coding strategy and fitness evaluation operation, which differs from that in MIHDE. The integer variable coding strategy of modified NHDE is used to represent which enzyme targets are selected to be regulated. Each target is then applied to solve the corresponding nonlinear programming problem and to sequentially compute the fitness of modified NHDE. Such a procedure is a parallel process to evaluate the objective function value of the MINLP problem. The evaluation operation in modified NHDE consists of two selection steps. The first selection step is a one-to-one competition that selects enzyme target for the next generation. The second step determines the best enzyme target in the population. However, the MIHDE algorithm directly uses the objective function in the MINLP problem as a fitness function for evaluating whether each selected target replaces its competitor or is rejected. Moreover, the convergence rate is low when MIHDE is applied to solve high-dimensional MINLP problems. The modified NHDE algorithm can overcome such drawbacks and is discussed in supporting information (S1 File).

## Results

### Design specifications

**Fig 4:**
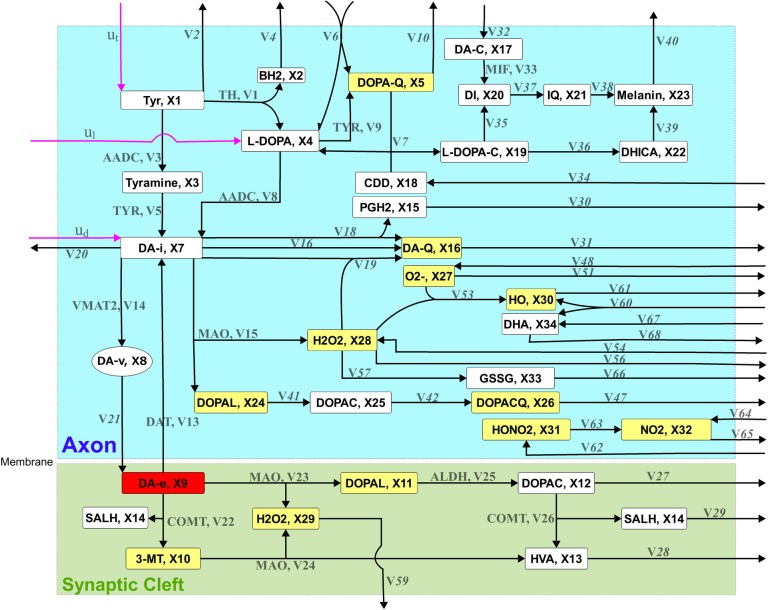
Schematic network diagram of the presynaptic dopamine metabolic pathway. The extracellular dopamine (DA-e) concentration of 400 (relative unit) under a healthy state (HS) was first obtained from the kinetic model. The concentrations of the metabolites for various deficiencies of VMAT2 and TH are listed in supporting information (S1 Table). DA-e is the therapeutic objective: the dopamine level as close to its healthy level as possible that the identified enzyme targets can achieve. Other metabolites of interest included toxic species, reactive oxygen species (ROS), and reactive nitrogen species (RNS). Toxic species considered in this model were dopaquinone (DOPA-Q), 3-methoxytyramine (3-MT), 3,4-dihydroxyphenylacetaldehyde (DOPAL), extracellular DOPAL (DOPAL-e), 3,4-dihydroxyphenylacetate quinone (DOPAC-Q), and dopamine quinone (DA-Q). ROS included superoxide (O_2_^−^), intracellular hydrogen peroxide (H_2_O_2_), extracellular H_2_O_2_ (H_2_O_2_-e), and hydroxyl radical (HO), whereas RNS included peroxynitrite (HO–NO_2_) and nitrogen dioxide (NO_2_). In this study, the toxic species, ROS, and RNS were considered the objectives for evaluating adverse effects.

In this study, the kinetic model of the nigrostriatal dopaminergic pathway reported in Qi *et al*. [36] was applied to formulate the fuzzy optimal drug design problem for identifying enzyme targets to remedy two types of enzymopathies caused by deficiencies of vesicular monoamine transporter 2 (VMAT2) and tyrosine hydroxylase (TH). This generalized mass action (GMA) model included 34 metabolites, 18 independent variables, and 68 target enzymes. The drug discovery problem had three external controls exerted to tyrosine, L-DOPA, and the intracellular dopamine, as shown in Fig 4. The detailed definition of the GMA model is expressed in supporting information (S2 File).

### Deficiencies of VMAT2

To illustrate the performance of the drug discovery problem, the first case study considered four different severities of enzymopathies: 50%, 70%, 90%, and 95% VMAT2 deficiencies (referred as VM50, VM70, VM90, and VM95, respectively). The enzyme activity for each deficiency was calculated using $\alpha_{VMAT2}^{DS}=(1-\delta /100)\alpha_{VMAT2}^{basal}$, where *δ* is the percentage deficiency for VMAT2. The DA-e concentration was zero for deficiencies >90%. First, we considered only the therapeutic effect objective for identifying enzyme targets for each deficiency level; the optimal results are shown in Table 1. The therapeutic objective could achieve 100% satisfaction grade for all cases. The activity of dopamine transporter (α_13_) decreased from 3.36 (relative unit) to the basal values of 1.57 and 0.52 for VM50 and VM70, respectively, indicating that only one enzyme target was sufficient to completely recover to the healthy state. This outcome also implies that α_13_ was downregulated to reduce the DA-e transport rate, which restores intracellular dopamine (DA-i). In addition, we computed the satisfactory grades for both the adverse and target variation scenarios in VM50 and VM70. The adverse effect yielded satisfactory scores for both VM50 and VM70, but the target variation effect for VM70 required more perturbation, indicating low satisfaction levels. For VM90 and VM95, two and three enzyme targets were required to fulfill the therapeutic objective; however, their corresponding adverse and target variation effect grades were less satisfactory.

**Table 1:** Optimal results for the drug discovery problems with different deficient conditions.

	Therapeutic effect				Therapeutic and adverse effects				Therapeutic, adverse and variation effects			
Disease	T*	A	V	Detected target	T*	A*	V	Detected target	T*	A*	V*	Detected target
VM50	1	1	0.636	α_13_	1	1	0.636	α_13_	0.839	0.839	0.926	α_15_
VM70	1	1	0.107	α_13_	1	1	0.107	α_13_	0.818	0.873	0.818	α_15_, α_60_
VM90	1	0.651	0.009	α_13_, α_15_	0.994	0.994	0	α_2_, α_13_, α_15_	0.697	0.749	0.697	α_13_, α_15_, α_60_
VM95	1	0.052	0.019	α_10_, α_13_, α_15_	1	1	0.13	α_6_, α_13_, α_15_, α_31_	0.574	0.611	0.574	α_13_, α_15_, α_16_, α_60_
TH50	1	0.975	0.958	α_56_	1	1	0.543	α_31_	0.999	0.974	0.974	α_42_
TH70	1	0.945	0.826	α_56_	1	1	0.426	α_9_	0.97	0.92	0.92	α_56_
TH90	1	1	0.329	α_2_, α_14_	1	1	0.334	α_2_, α_14_	0.851	0.998	0.851	α_14_, *u*_*t*_
TH100	1	0.929	0.087	*u*_*t*_, *u*_*l*_	0.951	0.951	0.105	*u*_*t*_, *u*_*l*_	0.651	0.588	0.588	α_2_, α_8_, *u*_*t*_, *u*_*l*_, *u*_*d*_

VM50 and VM95 reflect 50% and 95% deficiency of VMAT2, respectively, whereas TH50 and TH100 indicate 50% and 100% deficiency of TH, respectively. Furthermore, *u*_*t*_, *u*_*l*_, and *u*_*d*_ denote the external control for tyrosine, L-DOPA, and intracellular dopamine, respectively. α_*j*_ is the enzyme activity for the *j*^th^ reaction rate. T, A, and V denote the satisfaction grade for therapeutic, adverse, and variation effects, respectively.

* indicates the optimal solution.

We next considered the therapeutic and adverse effect objectives simultaneously for identifying the set of drug targets shown in the second column of Table 1. For VM50 and VM70, downregulation of α_13_ was sufficient for achieving 100% satisfaction level, which was similar to the aforementioned case. However, three and four enzyme targets were identified for VM90 and VM95, respectively, both showing satisfaction levels; however, their corresponding target variation effects were unsatisfactory.

**Fig 5:**
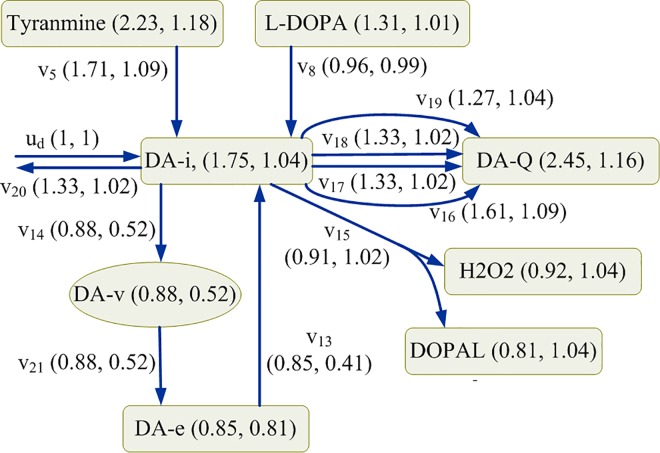
Steady-state material balances of the intracellular dopamine. 50% deficiency of VMAT2 is downregulated by α_15_ and α_13_, respectively. The first element in the parentheses indicates the fold change in the concentration and the flux, which is downregulated by α_15_, and the second element is the fold change downregulated by α_13_. Fold change is defined as the optimal regulated solution divided by its values in a healthy state.

Subsequently, we considered the therapeutic, adverse, and target variation effects simultaneously. VM50 yielded overall 83.9% satisfaction level despite α_13_ replacement by monoamine oxidase (α_15_) downregulation. Application of α_13_ to this case yielded 78.5%, 98.2%, and 78.5% satisfaction levels for the therapeutic, adverse, and target variation effects, respectively, providing an overall grade less than that observed for α_15_. The material balance equation on DA-i required four influxes and seven effluxes (Fig 5) to explain the aforementioned difference. In the case of downregulation of α_13_ (0.41-fold), *v*_14_ reduced the transport rate by 0.52 times, and the other fluxes changed insignificantly. Consequently, the concentrations of DA-i, DA-Q, H_2_O_2_, and DOPAL increased by 1.04, 1.16, 1.04, and 1.04 times, respectively. By contrast, in the case of downregulation of α_15_ (0.91-fold), *v*_14_ reduced the transport rate by 0.88 times; in addition, the other fluxes changed significantly: *v*_17_, *v*_18_, and *v*_20_ changed by a factor of 1.33, and *v*_19_ changed by a factor of 1.27. Hence, the concentrations of DA-i, DA-Q, H_2_O_2_, and DOPAL increased by a factor of 1.75, 2.45, 0.92, and 0.81, respectively. These different metabolic redistributions caused by the downregulation of α_13_ or α_15_, yielded different results for the therapeutic, adverse, and target variation effect objectives.

**Fig 6:**
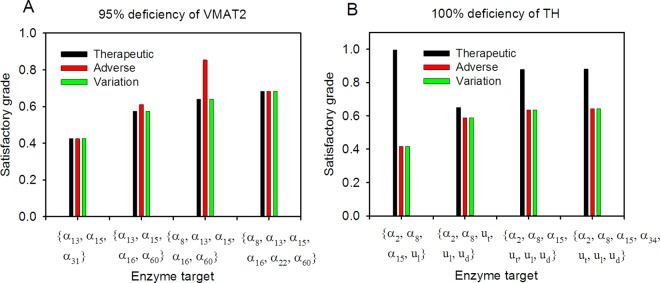
Number of enzyme targets for VMAT2 and TH. Optimal drug discovery considered with therapeutic, adverse, and variation effects simultaneously, at the specified number of enzyme targets. The left figure (A) is the case for 95% deficiency of VMAT2, and the right (B) for 100% deficiency of TH, whereas *u*_*t*_, *u*_*l*_, and *u*_*d*_ denote the external control for tyrosine, L-DOPA, and intracellular dopamine, respectively. Furthermore, α_*j*_ is the enzyme activity for the *j*^th^ reaction rate.

For VM70, VM90, and VM95, we required two, three, and four enzyme targets, respectively, to achieve the Pareto optimal design. From the results, we observed that Pareto optimal solutions followed the no free lunch theorem for optimization (*i*.*e*., a more severe dopamine deficiency requires more enzyme targets and lower satisfaction grades). Moreover, we compared objectives, namely therapeutic effect only and therapeutic effect plus the adverse effect, that could achieve 100% satisfaction level for the VM95 case. However, the overall satisfaction grade of 57.4% could only be realized when all three objectives (Table 1) were considered simultaneously. Furthermore, we compared the effects on the numbers of enzyme targets. We fixed the number of enzyme targets to three, five, and six and applied NHDE to solve each drug discovery problem. Optimal solutions for the aforementioned conditions are presented in Fig 6. The overall satisfaction grade obtained using three enzymes was less than that obtained using four enzymes. By contrast, the optimal grade obtained using five and six enzymes were higher than that obtained using four enzymes. Notably, the overall satisfaction grade was 64% when five enzyme targets were used, but the adverse effect was 85.4%.

### Deficiencies of TH

The second case study considered four severities of TH: 50%, 70%, 90%, and 100% deficiencies (referred as TH50, TH70, TH90, and TH100, respectively). Despite a complete TH deficiency (TH100), for HS, the DA-e concentration levels changed from 400 to 245.7 because DOPA decarboxylase converts L-DOPA to DA-i and is sequentially transported to the DA-e pool.

Following the procedures discussed in the first case study, we obtained the optimal enzyme targets for different design specifications and deficiencies (Table 1). For TH50, TH70, and TH90, using both one and two enzyme targets fulfilled the therapeutic and adverse effects when one or two objectives were considered in the drug discovery problem. Moreover, the overall satisfaction grade could reach 85.1% regulating with α_14_ and *u*_*t*_ when the therapeutic, adverse, and target variation effects were considered. However, for TH100, the overall satisfaction grade was 58.8% with five enzyme targets when the three objectives were considered simultaneously (Table 1). Furthermore, we compared the effects on the numbers of enzyme targets. We fixed the number of enzyme targets to four, six, and seven and applied NHDE to solve each drug discovery problem. Each optimal solution is presented in Fig 6. Although the therapeutic level could achieve nearly 100%, the overall satisfaction grade of 41.6% obtained with four enzymes was less than that obtained with five enzymes. By contrast, the optimal grades when using six and seven enzyme targets were higher than that obtained with five enzymes (Fig 6).

### Current Parkinson’s disease medications

Several prescription drugs and their corresponding enzyme targets are currently used for treating PD. The current medication are as following. The treatment of tyrosine intake control (u_t_) is a diet control. Madopar and Sinemet are a clinical remedy for providing L-DOPA (u_l_). Bromocriptine, Pergolide, Pramipexole and Ropinirole the prescription drugs for dopamine agonist (u_d_). Entacapone and Tolcapone are catechol-O-methyl transferase (COMT) inhibitors, which correspond to the rate constants of α_22_ and α_26_. Selegiline and Rasagiline are prescribed for inhibiting MAO activities (MAOI), which correspond to the rate constants of α_15_, α_23_, and α_24_. These current clinical drugs were respectively applied to treat PD caused by deficiencies of VMAT2 and TH in order to evaluate the therapeutic, adverse, and target variation effects; the computational results for various cases were listed in supporting information (S2 and S3 Tables). We observed that the current clinical drugs were unable to remedy diseases for 90 and 95% deficiencies of VMAT2, *i*.*e*. optimal solutions in the drug discovery problem for VM90 and VM95 were unable to be found. We found that MAOIs could remedy PD with the highest satisfaction grades among PD medications for VM50 and VM70 (S2 Table). Similarly, several deficiencies of TH were incapable of treatment by the current clinical drugs (S3 Table). However, for TH50, the current PD medications could achieve >90% overall satisfaction level. For TH70, MAOIs, COMT inhibitors and tyrosine intake control yielded >80% satisfaction grades.

In the case of VM50 (S2 Table), satisfactory therapeutic efficacy (>56%) could be achieved with the clinical drugs examined in this model. However, the satisfaction level of adverse effects varied from 22% to 80%. We investigated the concentrations of the neurotoxic species in VM50, to clarify the possible causes of neurotoxic and neuroprotective effects of the current medications for PD. Table 2 shows the concentrations of toxic, ROS and RNS species at healthy state (HS), their fold changes and the corresponding satisfaction grades of the adverse effect in VM50 and various treatments. Four neurotoxic species in VM50, namely DOPA-Q, DA-Q, HO, and HO–NO_2_, were higher than 1.2 fold change (or less than 0.94 satisfaction grade) to their levels in HS. L-DOPA, dopamine agonist, MAOI, and COMT inhibitors were respectively applied to treat the disease caused by VM50. The extracellular dopamine level was remedied from the fold change of 0.486 to 0.673 after treating L-DOPA. However, the concentrations of DOPA-Q, DA-Q, HO, and HO–NO_2_, increased about 8.0, 4.4, 2.5, and 2.3-folds, respectively, *i*.*e*. induced more neurotoxic species. We obtained the similar results by treating dopamine agonist, MAOI, and COMT inhibitors, respectively, as shown in Table 2. MAOI could achieve 100% therapeutic satisfaction grade and 80% adverse satisfaction grade for treating VM50.

**Table 2:** Concentrations (relative unit) of metabolites at healthy state, their concentration fold changes and their corresponding satisfaction grades of the adverse effect in VM50 and various treatments by current prescription drugs.

	Concentration	Fold change (*η*_*j*_(*x*_*j*_))					
Metabolite	HS	VM50/HS	L-DOPA/HS	DA-AG/HS	COMT/HS	MAOI/HS	Status
DA-e, *x*_9_	400	0.486(0.43)	0.673 (0.636)	0.806 (0.784)	0.735 (0.756)	1.0 (1.0)	Therapeutic
DOPA-Q, *x*_5_	5	1.512 (0.943)	7.962 (0.226)	5.429 (0.508)	3.192 (1.0)	2.634 (0.818)	Toxic
3-MT, *x*_10_	20	1.101 (0.989)	1.057 (0.994)	1.032 (0.996)	0.1 (1.0)	1.035 (0.996)	Toxic
DOPAL-e, *x*_11_	10	0.241 (1.0)	0.457 (1.0)	0.653 (1.0)	0.262 (1.0)	0.706 (1.0)	Toxic
DA-Q, *x*_16_	10	1.548 (0.939)	4.413 (0.621)	6.953 (0.339)	3.593 (0.712)	2.801 (0.8)	Toxic
DOPAL, *x*_24_	5	1.095 (0.989)	1.271 (0.97)	1.463 (0.949)	1.294 (0.967)	0.738 (1.0)	Toxic
DOPAC-Q, *x*_26_	10	1.127 (0.986)	1.446 (0.95)	1.672 (0.925)	1.41 (0.954)	0.757 (1.0)	Toxic
O_2_^−^, *x*_27_	5	1.182 (0.98)	1.857 (0.905)	2.042 (0.884)	1.613 (0.932)	0.921 (1.0)	ROS
H_2_O_2_, *x*_28_	5	1.13 (0.986)	1.543 (0.94)	1.694 (0.923)	1.422 (0.953)	0.872 (1.0)	ROS
H_2_O_2_-e, *x*_29_	2	0.763 (1.0)	0.854 (1.0)	0.915 (1.0)	0.344 (1.0)	0.97 (1.0)	ROS
HO, *x*_30_	2	1.287 (0.968)	2.471 (0.837)	3.001 (0.778)	2.074 (0.881)	0.758 (1.0)	ROS
HO-NO_2_, *x*_31_	2	1.24 (0.973)	2.301 (0.855)	2.553 (0.827)	1.862 (0.904)	0.961 (1.0)	RNS
NO_2_, *x*_32_	2	1.144 (0.984)	1.77 (0.914)	1.902 (0.9)	1.508 (0.943)	1.002 (1.0)	RNS

HS and VM50 denote as the healthy state and 50% VMAT2 deficiency, respectively. L-DOPA/HS, DA-AG/HS, COMT/HS and MAOI/HS are the concentration fold changes for treating L-DOPA, dopamine agonist, MAOI, and COMT inhibitors, respectively. *η*_*j*_(*x*_*j*_) in parentheses is the corresponding satisfaction grade of the adverse effect.

To remedy the most severe illnesses, namely VM95 and TH100, the NHDE algorithm was then applied for identifying a new set of formulated drugs, which included each current prescription drug with their detected targets. Table 3 lists the optimal detected targets combined with their prescription drugs. For VM95, the COMT inhibitors combined with four enzyme targets (α_13_, α_15_, α_31_, and α_61_) could achieve 62.6% satisfaction level, which was higher than the previous result obtained using four targets, as shown in the first row of Table 3. Thus, targets α_13_ and α_15_ were found in each medication. By contrast, for TH100, the MAOIs combined with five enzyme targets (α_2_, α_8_, *u*_*t*_, *u*_*l*_, and *u*_*d*_) achieved 63.7% satisfaction level, which was higher than the previous result obtained using five targets, as shown in the first row of Table 3. Thus, targets α_2_ and α_8_ were found in each medication.

**Table 3:** Optimal detected targets combined with their prescription drugs for the multiobjective drug discovery problem to remedy the most severe dopamine deficiency.

Disease	T*	A*	V*	Detected target	Specified target (Prescription drugs)
VM95	0.574	0.611	0.574	α_13_, α_15_, α_16_, α_60_	_—_
	0.425	0.493	0.425	α_13_, α_15_	u_t_ (Diet control)
	0.588	0.588	0.588	α_13_, α_15_, α_31_, α_60_	u_l_ (Madopar, Sinemet)
	0.605	0.605	0.605	α_13_, α_15_, α_31_, α_61_	u_d_ (Bromocriptine, Pergolide, Pramipexole, Ropinirole)
	0.626	0.626	0.626	α_13_, α_15_, α_31_, α_61_	α_22_, α_26_ (Entacapone, Tolcapone)
	0.576	0.611	0.576	α_13_, α_16_, α_60_	α_15_, α_23_, α_24_ (Selegiline, Rasagiline)
TH100	0.651	0.588	0.588	α_2_, α_8_, *u*_*t*_, *u*_*l*_, *u*_*d*_	_—_
	0.651	0.588	0.588	α_2_, α_8_, *u*_*l*_, *u*_*d*_	*u*_*t*_ (Diet control)
	0.651	0.588	0.588	α_2_, α_8_, *u*_*t*_, *u*_*d*_,	*u*_*l*_ (Madopar, Sinemet)
	0.651	0.588	0.588	α_2_, α_8_, *u*_*t*_, *u*_*l*_	*u*_*d*_ (Bromocriptine, Pergolide, Pramipexole, Ropinirole)
	0.503	0.682	0.508	α_2_, α_8_, *u*_*t*_, *u*_*l*_	α_22_, α_26_ (Entacapone, Tolcapone)
	0.637	0.637	0.637	α_2_, α_8_, *u*_*t*_, *u*_*l*_, *u*_*d*_	α_15_, α_23_, α_24_ (Selegiline, Rasagiline)

The current prescription drugs for PD treatment are shown in parentheses. VM95 reflect 95% deficiency of VMAT2 and TH100 indicate 100% deficiency of TH. Furthermore, *u*_*t*_, *u*_*l*_, and *u*_*d*_ denote the external control for tyrosine, L-DOPA, and intracellular dopamine, respectively. α_*j*_ is the enzyme activity for the *j*^th^ reaction rate. T, A, and V denote the satisfaction grade for therapeutic, adverse, and variation effects, respectively.

* indicates the optimal solution.

## Discussion

The main strategy in treating motor symptoms of PD is dopamine replacement. However, neuronal death and adverse effects were observed despite medical treatments. Several metabolites in the dopamine metabolism, including ROS, RNS, and toxic species, were speculated to be neurotoxic and might cause neurodegeneration in PD. Therefore, considering the therapeutic, adverse, and target variation effects simultaneously is essential for making a PD treatment plan. In the study, the concentration of DA-e in VM95 was lower than that in TH100, whereas VM95 showed high concentrations of neurotoxic metabolites, namely DOPA-Q, DA-Q, HO^.^, and HO^.^-NO_2_, compared with those observed in TH100. The results are compatible with the experimental findings that a reduction in VMAT2 levels causes a severe reduction in dopamine levels; elicits the nigrostriatal neurodegeneration, making neurons vulnerable to various toxic agents; and causes motor deficits [37–39]. When considering only the therapeutic objective, two and three enzyme targets were detected in TH100 and VM95, respectively, to completely restore the concentration of DA-e to the basal levels. However, after treatment, the concentrations of neurotoxic metabolites in VM95 were still substantially higher than those in TH100. The fuzzy multiobjective optimization approach was performed with a trade-off procedure for obtaining a compromise design to yield reduced therapeutic effect in order to improve adverse and target variation effects.

This study also aimed to reduce the target variation effect and minimize the number of enzyme targets. Reducing target variation effect leads to less perturbation in the enzyme activities, implying usage of low drug doses and subsequently minimizing the adverse effects. Furthermore, combination therapies are frequent in current PD treatments, and the simulation shows that multiple enzyme targets are detected in treatments for severe enzymopathies. However, drug combination may cause unexpected effects beyond those caused by an individual drug [40]. Consequently, the number of enzyme targets should be minimized when the therapeutic, adverse, and target variation effects are satisfied.

One of the pathological hallmarks of PD is the loss of dopaminergic neurons in the substantia nigra pars compacta; the hypothesis is that dopamine itself may be toxic through oxidative stress caused by auto-oxidation [41, 42]. Clinically, the precursor of dopamine, L-DOPA, is the most effective therapeutic agent for symptomatic relief of PD. However, chronic L-DOPA treatment might be harmful [43, 44], and L-DOPA could be neurotoxic [45, 46]. In addition, many in vitro studies have shown that L-DOPA and dopamine are cytotoxic [47, 48]. Gandhi *et al*. [5] reported that midbrain cell death increased in wide type and PINK1 gene knockout mice when the dopamine concentration increased. A complex of ROS production and calcium signaling were proposed to be the cause of dopamine induced cell death. Contrarily, the DATATOP study and other follow-up trials have demonstrated that MAOI delays the use of L-DOPA [6–9]. The computational results (Table 3, S2 and S3 Tables) confirmed the aforementioned clinical observations. The computation using MAOI yielded concentrations of most neurotoxic metabolites below the basal levels; however, concentrations of several neurotoxic metabolites elevated when using L-DOPA.

In the work of Qi *et al*. [36], gain analysis was used to aid the screening and selection of pharmacological therapies. In their simulation, increasing VMAT2 and MAO inhibition could elevate concentrations of extracellular dopamine. Furthermore, combined targeting of VMAT2 and MAO increased the extracellular dopamine and reduced the concentrations of toxic species. This method examined enzyme targets in turn, and failed to simulate all the combinations. In OPDD [21, 22], optimization search and decision making were separated into two stages. The first stage of the OPDD is to enumerate each enzyme to identify a set of candidate enzyme targets that fulfill the therapeutic objectives. The second stage of the OPDD is a posterior decision making determining a satisfactory target from the candidate enzyme targets. This method is less competent by reason of the manual process of decision making. In our work, we introduce a fuzzy multi-objective optimization approach to solve the enzyme target design problem. The problem is a unified optimization framework, in which the identification of enzyme targets is combined with multi-criteria decision-making. This proposed method can consider all the objectives simultaneously and identify the optimal enzyme targets for drug discovery efficiently.

It should be noted that there are limitations for the simulation of human metabolic disorders. The prediction accuracy of the computation depends on the accuracy of the mathematical model. Currently, the models of human metabolic networks are still under construction. According to the advances in computational biology, some algorithms, such as the machine learning methods for identification of multi-functional enzymes [49, 50], can be valuable for the construction of metabolic network. The results of our simulations may become more precise to experimental results when the models become more complete. In this work, we propose the method to act as a computer-aided design (CAD) of the drug discovery. This method maybe unable to identify the exact targets for new drugs but can narrow down the targets for further trial and error method. Eventually, the results of simulation should be verified with the results of experiment.

## Conclusions

A model-based drug target discovery problem is a decision-making problem, which can consider many criteria in the optimization formulation. This study introduced a fuzzy decision-making method for identifying enzyme targets of model-based drug discovery problems in presynaptic dopamine metabolic networks. This method combined several objectives in a unified framework to perform a trade-off procedure for obtaining a compromise design. In the case study, three fuzzy objectives, namely therapeutic, adverse, and target variation effects, and a number of targets were applied simultaneously for identifying enzyme targets. Moreover, the effects and adverse effects of current medications can be compared in this model to clarify the possible causes of neuroprotective and neurotoxic effects. Although the optimization approach is a trend in drug discovery, meaningful and exact modeling is the core facilitator of the desired outcome.

## Supporting Information

S1 File The detailed computational procedures. (DOCX)

S2 File List of dependent and independent variables for presynaptic dopamine metabolic model. (DOCX)

S1 Table The steady state concentrations of metabolites in healthy condition and various deficiencies of VMAT2 and TH. (DOCX)

S2 Table The therapeutic, adverse, and target variation effects remedied by the current clinical drugs to treat PD caused by deficiencies of VMAT2. (XLSX)

S3 Table The therapeutic, adverse, and target variation effects remedied by the current clinical drugs to treat PD caused by deficiencies of TH. (XLSX)
