# Supplementary material for: Fuzzy Decision Making Approach to Identify Optimum Enzyme Targets and Drug Dosage for Remedying Presynaptic Dopamine Deficiency
Source: PLoS One. 2016 Oct 13;11(10):e0164589. doi: 10.1371/journal.pone.0164589 (PMC5063375; doi:10.1371/journal.pone.0164589)
Supplement: S1 File — (DOCX) [file pone.0164589.s001.docx]

**Fuzzy Decision Making Approach to Identify Optimum Enzyme Targets and Drug Dosage for Remedying Presynaptic Dopamine Deficiency**

Kai-Cheng Hsu and Feng-Sheng Wang*

Department of Chemical Engineering

National Chung Cheng University

Chiayi 62102, Taiwan

Email: Kai-Cheng Hsu - [edwardfirst@gmail.com](mailto:edwardfirst@gmail.com); Feng-Sheng Wang*

[chmfsw@ccu.edu.tw](mailto:chmfsw@ccu.edu.tw)

**S1 Table.** Basic operations for the original differential evolution and nested hybrid differential evolution

| **Original DE** | **Nested HDE** |
| --- | --- |
| 1. Representation and initialization 2. Mutation 3. Crossover operation 4. Selection and evaluation 5. Repeat steps 2 to 4 | 1. Representation and initialization 2. Mutation with rounding operation 3. Crossover operation 4. Restriction operation 5. Selection and evaluation   Solve NLP problem for each combination of candidate targets  Compute fitness for each feasible design   1. Migration operation performed naturally or enforced if necessary 2. Repeat steps 2 to 6 |

**Modified NHDE algorithm for solving mixed-integer nonlinear programing problems**

## Algorithm

The fuzzy multiobjective drug discovery (FMDD) problem is expressed as following:

$\begin{matrix} \max_{\lambda,(\mathbf{x},\hat{\mathbf{\alpha}},\mathbf{u},\mathbf{z})\in\Psi} \left(\frac{Z^{UB}-\sum z_{j}}{Z^{UB}-1}\right)\lambda\\ \eta_{i}\left(x_{i}\right)\geq\lambda, i\in\Omega^{TE} \\ \eta_{j}\left(x_{j}\right)\geq\lambda, j\in\Omega^{AE} \\ \eta_{k}\left(\hat{\alpha}_{k}\right)\geq\lambda, k\in\Omega^{VE} \\ \eta_{l}\left(u_{l}\right)\geq\lambda,l\in\Omega^{VE} \\ \text{Equality constraints:} \\ \left\{\begin{matrix} \text{Kinetic model:} \\ \sum_{j=1}^{n_{v}} N_{ij}v_{j}+\sum_{j=1}^{n_{u}} B_{ij}u_{j}=0,i\in\Omega^{Species} \\ w_{j}=\hat{\alpha}_{j}+\sum_{k=1}^{n_{v}} g_{jk}y_{k},j\in\Omega^{rxn} \\ v_{j}=\exp\left(w_{j}\right)\text{ (nonlinear equation)} \\ x_{i}=\exp\left(y_{i}\right)\text{ (nonlinear equation)} \\ \\ \text{Disease restriction:} \\ \hat{\alpha}_{j}=\hat{\alpha}_{j}^{DS},j\in\Omega^{DS} \end{matrix}\right. \\ \text{Inequality constraints:} \\ \left\{\begin{matrix} x_{i}^{LB}\leq x_{i}\leq x_{i}^{UB}; i\in\Omega^{Species} \\ z_{j}\hat{\alpha}_{j}^{LB}+(1-z_{j})\hat{\alpha}_{j}^{basal}\leq\hat{\alpha}_{j}\leq z_{j}\hat{\alpha}_{j}^{UB}+(1-z_{j})\hat{\alpha}_{j}^{basal};j\in\Omega^{VE} \\ z_{k}u_{k}^{LB}+(1-z_{k})u_{k}^{basal}\leq u_{k}\leq z_{k}u_{k}^{UB}+(1-z_{k})u_{k}^{basal};k\in\Omega^{VE} \end{matrix}\right. \end{matrix}$ (S1)

The maximizing decision problem (S1) is a mixed-integer nonlinear programming (MINLP) problem, which is rewritten as a general optimization problem with constraints.

$\begin{matrix} \max_{\mathbf{x,z}} f\left(\mathbf{x},\mathbf{z}\right) \\ \text{subject to} \\ h_{i}\left(\mathbf{x},\mathbf{z}\right)=0,i=1,...,n_{h} \\ g_{j}\left(\mathbf{x},\mathbf{z}\right)\leq0,j=1,...,n_{g} \end{matrix}$ (S2)

The problem (S2) is converted into the penalty problem (S3) to be solved by the modified NHDE.

$\max_{\mathbf{x,z}} F\left(\mathbf{x},\mathbf{z}\right)=\max_{\mathbf{x,z}} \left\{f\left(\mathbf{x},\mathbf{z}\right)-\gamma\left(\sum_{i=1}^{n_{h}} h_{i}^{2}\left(\mathbf{x},\mathbf{z}\right)+\sum_{j=1}^{n_{g}} \left\langle g_{j}\left(\mathbf{x},\mathbf{z}\right)\right\rangle_{+}^{2}\right)\right\}$ (S3)

The basic operations of modified NHDE are shown in Tab. S1-1 or equivalent to S1-1 Fig. NHDE is a parallel direct search algorithm that utilizes a population of *N_p_* individuals to find an optimal solution. The initialization process randomly generates *N_p_* individuals to cover the entire search space uniformly. This representation is similar to a set-based reaction discussed in [1]. Without loss of generality, we assume that all rate constants are arranged in an ordered reaction set and then each individual consists of a set of regulated rate constants encoded by the order of their appearance in the reaction set. This representation is similar to the set-based reactions discussed in [1] and is shown in S1-2 Fig.

**S1-1 Table.** Basic operations for the original differential evolution and modified NHD algorithm

| **Original DE** | **Modified NHDE** |
| --- | --- |
| 1. Representation and initialization 2. Mutation 3. Crossover operation 4. Selection and evaluation 5. Repeat steps 2 to 4 | 1. Representation and initialization 2. Mutation with rounding operation 3. Crossover operation 4. Restriction operation 5. Selection and evaluation    1. Solve NLP problem for each candidate targets    2. Compute fitness for each feasible design 6. Migration operation performed naturally or enforced if necessary 7. Repeat steps 2 to 6 |

**S1-1 Fig.** Flowchart of the modified algorithm for nested hybrid differential evolution

$$\begin{matrix} \begin{matrix} \begin{matrix} \text{ }z_{1} & z_{2} \end{matrix} & \begin{matrix} z_{3} & z_{4} \end{matrix} & \begin{matrix} z_{5} & z_{6} \end{matrix} & \begin{matrix} z_{7} & z_{8} \end{matrix} \end{matrix} \\ \left\{\begin{matrix} \text{Indiv}1 & [1 & 3 & 5] \\ \text{Indiv}2 & [2 & 2 & 5] \\ \vdots& \vdots& \vdots& \vdots\\ \text{Indiv}N_{p} & [1 & 2 & 4] \end{matrix}\right.\text{ }\Rightarrow\text{ }\left\{\begin{matrix} \begin{matrix} 1\text{ } & 0\text{ } \end{matrix} & \begin{matrix} 1\text{ } & 0\text{ } \end{matrix} & \begin{matrix} 1\text{ } & 0\text{ } \end{matrix} & \begin{matrix} 0\text{ } & 0\text{ } \end{matrix} \\ \begin{matrix} 0\text{ } & 1\text{ } \end{matrix} & \begin{matrix} 0\text{ } & 0\text{ } \end{matrix} & \begin{matrix} 1\text{ } & 0\text{ } \end{matrix} & \begin{matrix} 0\text{ } & 0\text{ } \end{matrix} \\ \begin{matrix} \vdots\text{ } & \vdots\text{ } \end{matrix} & \begin{matrix} \vdots\text{ } & \vdots\text{ } \end{matrix} & \begin{matrix} \vdots\text{ } & \vdots\text{ } \end{matrix} & \begin{matrix} \vdots\text{ } & \vdots\text{ } \end{matrix} \\ \begin{matrix} 1\text{ } & 1\text{ } \end{matrix} & \begin{matrix} 0\text{ } & 0\text{ } \end{matrix} & \begin{matrix} 1\text{ } & 0\text{ } \end{matrix} & \begin{matrix} 0\text{ } & 0\text{ } \end{matrix} \end{matrix}\right. \end{matrix}$$

**S1-2 Fig. Representation of Individuals.** Each individual consists of a set of regulated rates reactions encoded by the order of their appearance in the reaction set.

The mutation operator of NHDE adopted from DE was an essential component compared with other evolutionary algorithms. Different from conventional evolutionary algorithms, the mutation operation of DE/NHDE uses the difference between two or four randomly chosen individuals as an evolutionary direction. The *i*^th^ mutant individual (**z***^G^*)*_i_* in generation *G* is obtained through the difference of two or four random individuals as expressed in the following form:

$(\mathbf{z}^{G})_{i}=\text{INT}\left\{(\mathbf{z}^{G})_{p}+\rho^{G}\left[(\mathbf{z}^{G})_{j}-(\mathbf{z}^{G})_{k}+(\mathbf{z}^{G})_{l}-(\mathbf{z}^{G})_{m}\right]\right\},i=1,...,N_{p}$ (S4)

where random indices $j,k,l,m\in\{1,\ldots,N_{p}\}$ are mutually different. The operator INT in Equation (S3) is used to rounding the real vector into the integer vector. In DE, the mutation factor *ρ^G^* is tuned between zero and one by the user. This factor is used to control the step length along the searching direction. A random mutation factor was used in NHDE to obtain more diversified individuals. NHDE also implants an additional mutation strategy that applying a linear crossover for the *i*^th^ individual and the best individual (**z***^G^*)*_b_* to generate the parent individual. The parent individual is therefore expressed as follows:

$(\mathbf{z}^{G})_{p}=\rho_{p}^{G}(\mathbf{z}^{G})_{b}+(1-\rho_{p}^{G})(\mathbf{z}^{G-1})_{i}$ (S5)

where the factor *ρ_p_^G^* is a real random number ranges between zero and one, and (**z***^G^*^-1^)*_i_* indicates the *i*^th^ mutant individual in the previous generation. The mutation operation may cause the mutant individual escape from the search domain. If a mutant individual is outside the search domain, then it is replaced within its lower bound and upper bound so that each individual was restricted on the search domain.

The core procedure of the NHDE algorithm is the “selection and evaluation” operation, which are different to MIHDE. The evaluation operation in NHDE consisted of two selection steps. The first selection operation was a one-to-one competition that determined which trial individuals would survive in the next generation. The next step selected the optimal individual in the population. The MIHDE algorithm directly uses the objective function in MINLP problems as a fitness function for evaluating whether each selected therapeutic agent replaces its competitor or is rejected. However, the convergence rate is low when MIHDE is applied to solve high-dimensional MINLP problems. Suppose that an offspring has the exact integer part of the optimal solution but an inexact real part. Although only one real variable of the individual is different from the optimal solution, the fitness is still quite bad. Consequently, the offspring should be rejected from the evaluation so that MIHDE may converge to obtain the optimal solution.

The NHDE algorithm can overcome such a drawback. Each operation in NHDE is performed only for the integer variables; in other words, the enzyme targets are selected in advance and are applied to the drug design problem to evaluate the design specifications. The selection and evaluation operation for NHDE involves two additional steps. The first evaluation step is to solve each nonlinear programming (NLP) problem produced from the maximizing decision problem (Eq.S1) for each target candidate. A global design can be achieved when the optimal solution form the NLP problem is obtained because the derived problem is a convex optimization problem for the given enzyme targets. By contrast, the fitness should be accompanied by a penalty value for infeasible solutions. Therefore, each fitness of NHDE is computed for selecting therapeutic agents for the next step.

Similar to conventional evolutionary algorithms, a crossover operation was employed to increase the local population diversity. DE/HDE utilized the difference of two mutually independent individuals as a searching direction toward an optimal solution. All individuals can quickly cluster together and superior individuals cannot be generated through mutation and crossover operations. The migration operation of NHDE is used to help all individuals escape from this local cluster. This migration operation is performed only if the measure of population diversity fails to satisfy the desired tolerance. Lin et al. [2] proposed the population diversity degree ζ to check whether the migration operation should be performed. In order to define the degree of population diversity, the gene diversity index at the *G* generation is introduced:

$dz_{ji}=\left\{\begin{matrix} 0, \text{if} z_{ji}^{G}=z_{jb}^{G}, j=1,...,n;i=1,...,N_{p};i\neq b \\ 1, \text{otherwise,} \end{matrix}\right.$ (S6)

where *z_jb_^G^* are the *j^th^* gene of the best individual at the *G^th^* generation, *dz_ji_* is the gene diversity index. According to the definition of gene diversity index, we set the *j*^th^ gene diversity index for the *i*^th^ individual to zero if this gene clusters around the best gene. The population diversity degree *ζ* is defined as the ratio of total gene diversities to total number of genes except that of the best individual. We therefore have the population diversity degree:

$\zeta=\frac{\sum_{i=1,i\neq b}^{N_{p}} \sum_{j=1}^{n} dz_{ji}}{n(N_{p}-1)}$ (S7)

The value of population diversity degree ranges between zero and one. A value of zero implies that all of the genes are clustered around the best individual. On the other hand, a value of one indicates that current candidate individuals are a completely diversified population. The desired tolerance for population diversity is assigned by the user. A tolerance value of zero implies that the migration operation in NHDE is switched off, and one implies that the migration operation is performed at every generation. Consequently, the user can set a tolerance value for population diversity degree, $\varepsilon\in\left[0,1\right]$. If *ζ* is smaller than *ε*, then NHDE performs migration operations to regenerate a new population in order to escape from a local point; otherwise, NHDE suspends the migration operation and maintains a constant search direction toward a target.

**References**

1. Patil K, Rocha I, Forster J, Nielsen J: **Evolutionary programming as a platform for in silico metabolic engineering**. *BMC Bioinformatics* 2005, **6**(1):308.
2. Lin YC, Wang FS, Hwang KS: **A Hybrid Method of Evolutionary Algorithms for Mixed-Integer Nonlinear Optimization Problems**, in *Proceedings of the 1999 IEEE Congress on Evolutionary Computation (CEC 99)*, Washington D. C., 1999 pp. 2159-2166.
