# Supplementary material for: Fuzzy Decision Making Approach to Identify Optimum Enzyme Targets and Drug Dosage for Remedying Presynaptic Dopamine Deficiency
Source: PLoS One. 2016 Oct 13;11(10):e0164589. doi: 10.1371/journal.pone.0164589 (PMC5063375; doi:10.1371/journal.pone.0164589)
Supplement: S1 Table — (DOCX) [file pone.0164589.s003.docx]

**Fuzzy Decision Making Approach to Identify Optimum Enzyme Targets and Drug Dosage for Remedying Presynaptic Dopamine Deficiency**

Kai-Cheng Hsu and Feng-Sheng Wang*

Department of Chemical Engineering

National Chung Cheng University

Chiayi 62102, Taiwan

Email: Kai-Cheng Hsu - [edwardfirst@gmail.com](mailto:edwardfirst@gmail.com); Feng-Sheng Wang* - [chmfsw@ccu.edu.tw](mailto:chmfsw@ccu.edu.tw)

**Supplementary file S1_Table:**

The concentrations of metabolites in the steady state were computed based on the governed model by using various rate constants. The metabolite concentrations were computed according to deficiencies of VMAT2 and TH. The first case study considered four different severities of enzymopathies: 50%, 70%, 90%, and 95% VMAT2 deficiencies (referred as VM50, VM70, VM90, and VM95, respectively). The second case study considered four severities of TH: 50%, 70%, 90%, and 100% deficiencies (referred as TH50, TH70, TH90, and TH100, respectively).

**S1 Table.** Concentrations (relative unit) of metabolites at healthy and eight pathological states. HS is the healthy state, 50%, 70%, 90%, and 95% VMAT2 deficiencies referred as VM50, VM70, VM90, and VM95, respectively, and 50%, 70%, 90%, and 100% TH deficiencies referred as TH50, TH70, TH90, and TH100, respectively.

| Metabolite | HS | VM50 | VM70 | VM90 | VM95 | TH50 | TH70 | TH90 | TH100 | Objective |
| --- | --- | --- | --- | --- | --- | --- | --- | --- | --- | --- |
| Extracellular dopamine (x_9_) | 400 | 194.42 | 101.34 | 0 | 0 | 387.53 | 372.91 | 328.01 | 245.7 | Therapeutic |
| Dopaquinone (x_5_) | 5 | 7.56 | 9.5 | 13.26 | 23.42 | 7.28 | 10.53 | 24.41 | 67.35 | Toxic |
| 3-Methoxytyramine (x_10_) | 20 | 22.03 | 23.58 | 35.5 | 12.69 | 20.09 | 20.21 | 20.58 | 21.4 | Toxic |
| Extracellular DOPAL (x_11_) | 10 | 2.41 | 0.66 | 0 | 0 | 9.39 | 8.71 | 6.76 | 3.82 | Toxic |
| Dopamine quinone (x_16_) | 10 | 15.48 | 19.87 | 28.95 | 57.49 | 8.99 | 7.93 | 5.29 | 2.26 | Toxic |
| Intracellular DOPAL (x_24_) | 5 | 5.48 | 5.76 | 6.2 | 7.06 | 4.92 | 4.81 | 4.47 | 3.81 | Toxic |
| DOPAC quinone (x_26_) | 10 | 11.27 | 12.05 | 13.32 | 15.93 | 9.74 | 9.43 | 8.48 | 6.73 | Toxic |
| Superoxide O_2_^−^ (x_27_) | 5 | 5.91 | 6.49 | 7.46 | 9.55 | 4.76 | 4.49 | 3.72 | 2.42 | ROS |
| Intracellular hydrogen peroxide H_2_O_2_ (x_28_) | 5 | 5.65 | 6.05 | 6.71 | 8.06 | 4.84 | 4.65 | 4.1 | 3.11 | ROS |
| Extracellular hydrogen peroxide H_2_O_2_ (x_29_) | 2 | 1.53 | 1.26 | 0.89 | 0.32 | 1.97 | 1.94 | 1.84 | 1.65 | ROS |
| hydroxyl radical HO^-^ (x_30_) | 2 | 2.57 | 2.97 | 3.67 | 5.4 | 1.87 | 1.72 | 1.33 | 0.76 | ROS |
| Peroxynitrite NO_3_^-^ (x_31_) | 2 | 2.48 | 2.8 | 3.36 | 4.66 | 1.87 | 1.74 | 1.36 | 0.79 | RNS |
| Nitrogen dioxide NO2 (x_32_) | 2 | 2.29 | 2.48 | 2.8 | 3.55 | 1.92 | 1.83 | 1.6 | 1.21 | RNS |
